# Isolation of inflammatory cells from rat brain tissue after stroke

**DOI:** 10.1186/2040-7378-4-20

**Published:** 2012-10-02

**Authors:** Karoline Möller, Tobias Stahl, Johannes Boltze, Daniel-Christoph Wagner

**Affiliations:** 1 Fraunhofer Institute for Cell Therapy and Immunology, Leipzig, Germany; 2 Department of Veterinary Anatomy, University of Leipzig, Leipzig, Germany; 3 IDT Biologika GmbH, Dessau-Rosslau, Germany; 4 Translational Centre for Regenerative Medicine, University of Leipzig, Leipzig, Germany

**Keywords:** Stroke, Inflammation, Flow cytometry, Animal models

## Abstract

The pathophysiology of sterile inflammation following focal ischemic stroke is complex and not fully understood, but there is growing evidence that it offers several therapeutic options beyond the hitherto existing treatment strategies. The identification and quantification of infiltrating inflammatory cells in animal models of stroke is crucial both for understanding post-stroke inflammation and for drug target identification. Multicolor flow cytometry plays an important role in determining subtypes and quantity of leukocytes that infiltrate the brain tissue after stroke. Until now, most investigations have been performed in mice, most likely due to a significantly broader spectrum of disposable antibodies and available knockout models. Here, we introduce a specific and reproducible method to isolate leukocytes from rat brain specimen in the context of brain ischemia to ultimately allow multi-dimensional flow cytometric characterization and further downstream methods such as cell-subtype sorting and molecular biological approaches.

## Background

Cerebral stroke is still one of the main causes of death and acquired disabilities worldwide. The sole established therapeutic option is thrombolysis, which is severely limited by a narrow time window and several contraindications [1]. Cumulatively, only 5 - 13% of stroke patients benefit from clot lysis [2,3] whereas the remaining survivors have to be content with symptomatic and preventive treatments. The situation is even worse for patients suffering from hemorrhagic stroke where no causal treatment is available. Hence, numerous preclinical and clinical trials have been performed to test novel treatment options to ultimately reach more stroke patients [4].

Amongst others, the modulation of inflammatory responses became a promising approach to treat cerebral ischemic and hemorrhagic stroke even days beyond the time window for thrombolysis [5]. The sterile inflammation after stroke was initially considered to be merely adverse and many pharmacological agents have been proposed to block this event [6]. However, recent evidence indicates a much more complex and partly conflicting picture [7]. Mostly by employing different knock-out mouse models, it has been emphasized that, in the early phase after stroke, T-cells act primarily detrimental [8,9] whereas B-cells seem to rather protect the injured brain [10]. The role of regulatory T-cells is still controversially discussed [11,12] and scarce knowledge is available about specific Th1, Th2 and Th17 responses [13] or the possible development of post-stroke autoimmunity and its sequelae [14].

Hence it is clear that the identification and quantification of infiltrating inflammatory cells in animal models of stroke is critical for understanding the complex processes of post-stroke inflammation and for the identification of novel therapeutic targets. Multicolour flow cytometry has turned out to be the gold standard to achieve this goal since it allows the simultaneous identification and quantification of several immune cell subtypes without the need to bias the system by in vivo staining or genetic manipulations [15].

Most work in the field has been done in mouse models of cerebral stroke [9,10,15,16], particularly due to a broader availability of antibodies and the possibility to use knockout models. However an important part of stroke research and therapy development is done in rats, since these animals allow sophisticated functional testing which is still considered to be the most important surrogate for therapeutic efficacy in stroke research. The isolation of inflammatory cells from rat brain tissue has been described earlier [17], but low cell yields may limit further analyses and necessitate pooling of biological replicates [15]. We therefore introduce a step-by-step protocol that was specifically developed to isolate high numbers of CD45-positive cells from ischemic rat brain tissue. The obtained cell suspensions can be analyzed by multi-dimensional flow cytometry or can be further enriched by immunoselection to ultimately perform highly specific downstream analyses.

### Materials

All quantities refer to tissue of one rat brain hemisphere.

1. Phosphate buffered saline (PBS) without CaCl_2_ and MgSO_4_, pH 7.4

2. Hanks balanced salt solution (HBSS; H9269, Sigma, Taufkirchen, Germany)

3. Modified HBSS, without CaCl_2_ and MgSO_4_ (H9394, Sigma)

4. Collagenase I-A (stock solution 20 mg/ml; C2674, Sigma)

5. DNase I (stock solution 2000 U/ml in PBS; 11284932001, Roche, Mannheim, Germany)

6. Percoll (17-0891-01, GE Healthcare, Munich, Germany)

7. NaCl 1.5 M

8. Fetal bovine serum (FBS; heat inactivated; PAN-Biotech, Aidenbach, Germany)

### Reagent setup

1. Density Gradient

• Prepare stock isotonic Percoll (SIP) by mixing nine parts of Percoll with one part NaCl 1.5 M.

• Dilute SIP to lower densities (80%, 38% and 21%) by adding appropriate volumes of HBSS (Ca^2+^/Mg^2+^ free) containing 3% of fetal bovine serum.

2. Digestion buffer (7.5 ml per brain hemisphere)

• Add each 375 μl of Collagenase I-A stock solution (final concentration 1 mg/ml) and DNase I stock solution (final concentration 100 U/ml) to 6.75 ml HBSS.

3. Washing buffer (for 30 ml)

• Mix 1.5 ml of DNase I stock solution (final concentration 100 U/ml) and 900 μl of fetal bovine serum with 27.6 ml HBSS.

### Laboratory equipment

1. Euthanasia chamber (CO_2_)

2. Equipment for transcardial perfusion (Pump, tubes and needle)

3. Dissecting set (Metzenbaum scissors curved and straight, standard pattern forceps, tissue forceps, hemostats curved and straight)

4. Polypropylene falcon conical tubes 15 ml and 50 ml

5. Falcon cell strainer 40 μm (BD352340, BD Biosciences, Heidelberg, Germany)

6. Scalpel or razor blade

7. Petri dishes

8. PVC serum pipettes 150mm (612361, Greiner Bioscience, Frickenhausen, Germany)

9. Glass pasteur pipettes 150mm (4518, Carl Roth, Karlsruhe, Germany; with modified blunt openings of two different widths, adjustment is done by fire polishing to diameters of 0.5 mm and 1 mm)

10. Pipetting aid

11. MACSmix tube rotator (130-090-753, Miltenyi Biotec, Bergisch Gladbach, Germany) or equivalent system for heat incubation

## Methods

All animal experiments must conform to according standards for animal care (e.g. the Guide for the Care and Use of Laboratory Animals published by the US National Institutes of Health, NIH Publication No. 85–23, revised 1996) and have to be approved by the appropriate authority.

Exemplarily, permanent right middle cerebral artery occlusion (MCAO) or sham surgery was induced in male spontaneously hypertensive rats (SHR, aging 12 weeks; Janvier, Le Genest-St-Isle, France) as described earlier [18,19]. Animals were sacrificed either 24h or 96h after surgery.

### Perfusion and sampling

**Figure 1:**
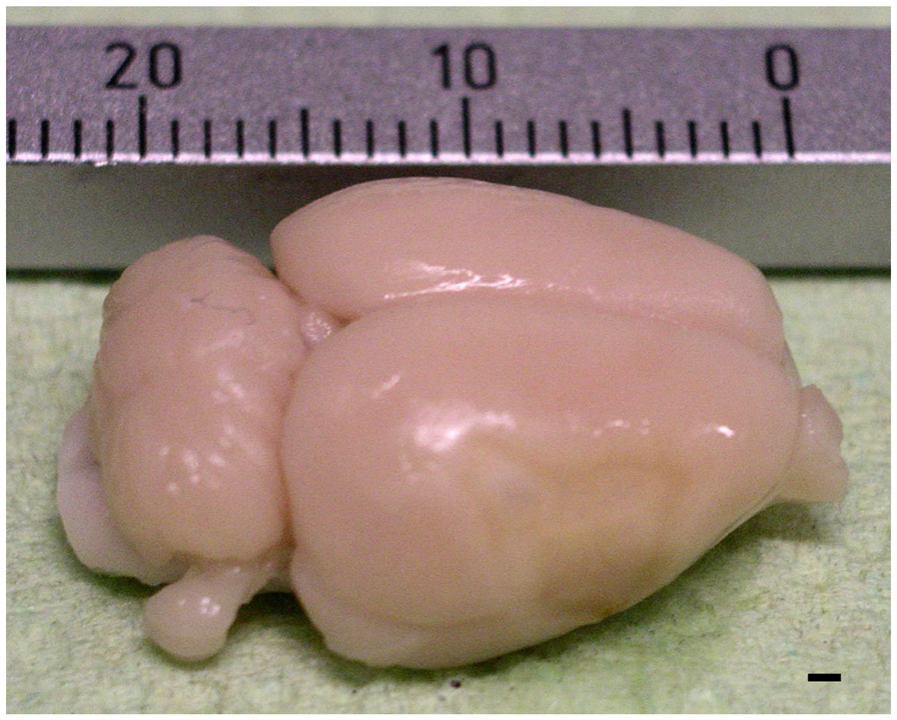
**Image of a representative rat brain showing a focal ischemic lesion within the right middle cerebral artery territory. **Both medulla oblongata and bulbus olfactorius was dissected before the brain was carefully removed from the cranium. After removal of the meninges and the cerebellum, brains were halved along the interhemispheric fissure and both hemispheres were further processed separately. Scale bar = 1 mm.

1. Animals are deeply anesthetized (e.g. with ketamine ketamine hydrochloride (100 mg/kg) and xylacine (10 mg/kg)) and sacrificed by CO_2_ exposition.

2. Transcardial perfusion with 200 mL PBS (4°C), decapitation, dissection of brain and removal of meninges, cerebellum and bulbus olfactorius (Figure 1).

3. Separation of hemispheres using a razor blade. Hemispheres can be kept short-term (< 45 minutes) in HBSS on ice.

4. Single hemispheres are mechanically dissociated by chopping the tissue approximately 120 times with a razor blade. Meanwhile, tissue must be moistened with 1mL of digestion buffer to prevent drying.

### Enzymatic dissociation (37°C)

1. Obtained cell suspension is mixed with the remaining 6.5 mL digestion buffer and transferred to a 15 mL tube using a PVC serum pipette

2. Suspension is then incubated under slow continuous rotation at 37°C for 45 minutes. The incubation is stopped by two additional mechanical trituration steps: after 15 minutes the suspension is slowly pipetted up and down (10 times) with a PVC serum pipette; after 35 minutes the suspension is completely dissociated by pipetting 10 times up and down with each of the two firepolished glass pipettes, using the wider tipped first.

Caution: Mechanical dissociation is crucial for obtaining optimal final cell yield and viability. It is essential to pipette slowly to avoid formation of bubbles. The edges of glass pipettes should be rounded.

3. Suspension is then filtered through a 40μm cell strainer, rinsed with 15 ml washing buffer and pelleted at 300xg for 8 minutes at room temperature. The supernatant is aspirated carefully, and the washing and centrifugation steps are subsequently repeated.

### Density gradient centrifugation (Room temperature)

Percoll should be used at room temperature to prevent cell clumping. For further details of Percoll handling and setting up of gradients please refer to the according manual (http://www.gelifesciences.com).

**Figure 2:**
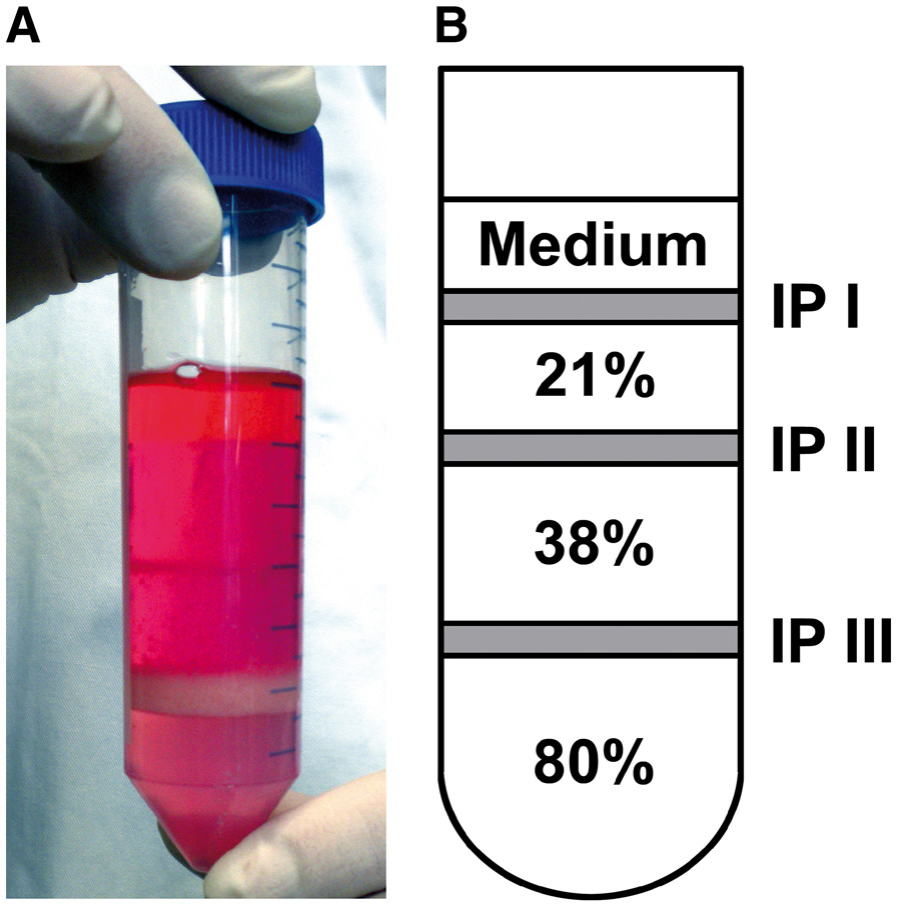
**(A) Representative picture of the prepared gradient and (B) schema of the composition of the gradient after centrifugation. **IP: interphase.

1. The cell pellet is resuspended in 10 ml of 80% SIP and transferred to a 50 ml tube. Using a 10 ml serum pipette, 10 ml of 38% SIP are slowly layered on top of the cell suspension followed by another 10 ml of 21% SIP. Finally the gradient is covered with 5 ml of HBSS containing 3% FBS (Figure 2).

Caution: It is crucial to perform pipetting in a slow, consistent manner. The tip of the pipette should be positioned at the inner wall of the tube just above the subjacent layer, but without touching it. This assures discrete layers without swirling at the interphases.

2. Centrifugation is performed at 480xg for 35 minutes at 18°C with minimal acceleration and no brake using a swinging bucket rotor.

3. The top layer and the first interphase (containing myelin and debris) as well as the second layer and second interphase (containing mostly CD45-negative cells and low amounts of microglia) are gently aspirated and discarded (Figure 2).

4. The fraction accumulated at the third interphase (Figure 2) is collected into a fresh 50 ml tube using a PVC serum pipette.

5. Cells are diluted to a volume of 30 ml by adding HBSS (Ca/Mg free) containing 3% of FBS and gently tossed.

6. Centrifugation is done at 300xg for 8 minutes, supernatant is discarded and the resulting cell pellet is resuspended in a small volume of HBSS buffer, transferred to a new 15 ml tube and washed again.

Caution: These washing steps are crucial since the sufficient removal of Percoll significantly contributes to the quality of the final cell sample.

7. The resulting cell pellet is resuspended in an appropriate amount of FACS-buffer (Ca^2+^/Mg^2+^ free PBS containing 3% FBS) for quantification, antibody labelling and adjacent analysis. Suspension is kept at 4°C.

### Quantification and processing for flow cytometry (4°C)

1. Isolated immune cells are stained with trypanblue and quantified in a hemacytometer. Viability is calculated by the ratio of trypanblue positive and negative cells.

2. According to cell yield and flow requirements aliquots of up to 1x10^5^ cells are adjusted in 100 μl cold (4°C) FACS-buffer.

3. Prior to antibody labeling, cells are incubated with FC-blocking reagent (purified anti-rat CD16) at 4°C for 20 minutes to prevent unspecific binding. Antibody panels for brain immune cell characterization used in our experiments consist of up to 8 fluorochrome conjugated antibodies and essentially contain the pan-leukocyte-marker anti-CD45.

### Outcome

**Figure 3:**
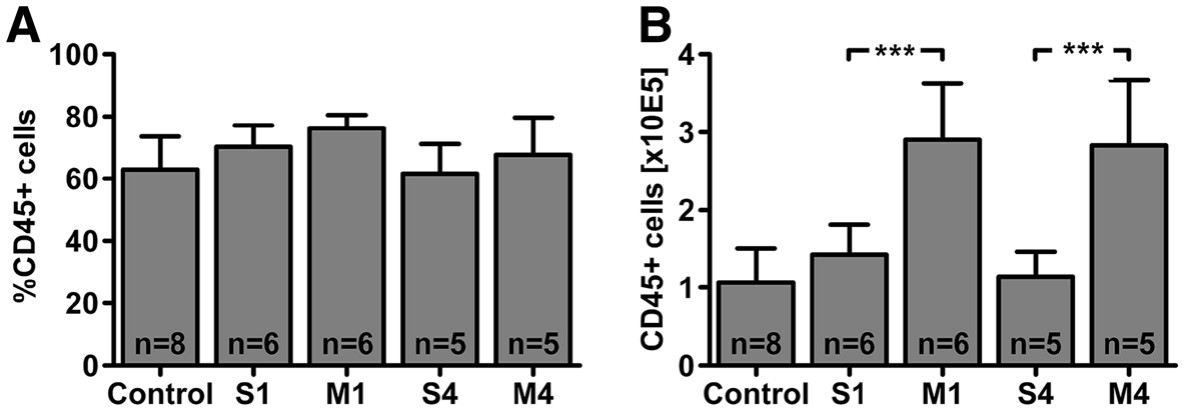
**(A) The proportion of CD45**-**positive cells within the lifegate was comparable between the experimental groups. **(**B**) Absolute CD45-positive cell yield of the right hemisphere after different experimental conditions. The absolute number of cells was comparable in control animals and animals subjected to sham surgery (S1: day 1, S4: day 4). Permanent middle cerebral artery occlusion (MCAO) caused a doubling of CD45-positive cells both at day 1 (M1) and day 4 (M4) after stroke onset (***p<0.001 by one-way ANOVA followed by Bonferroni post hoc test). Data is shown as means ± SD.

**Figure 4:**
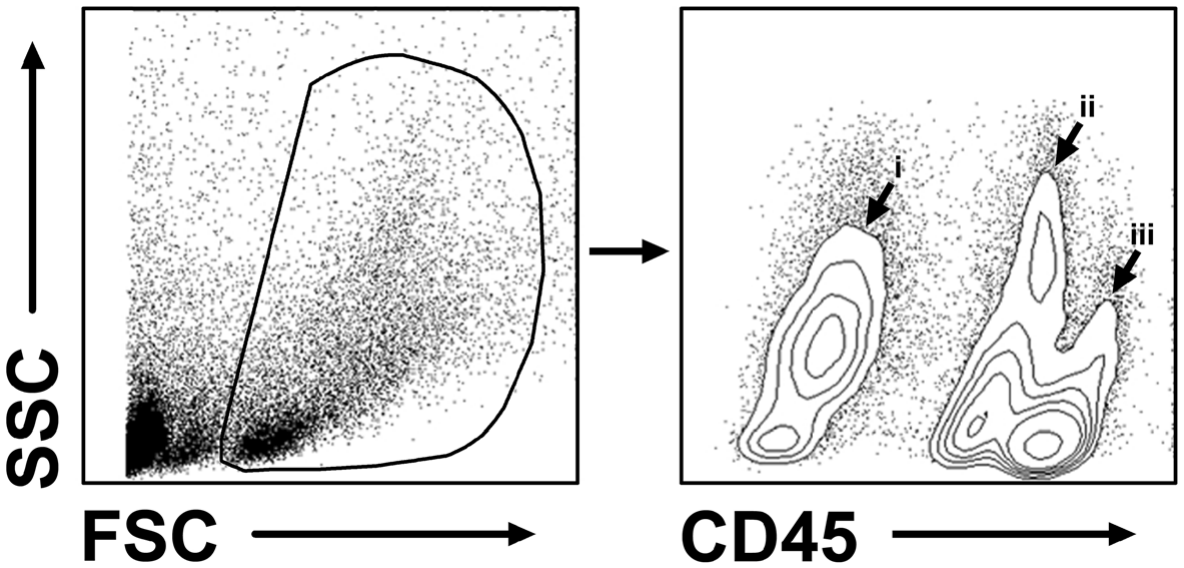
**Exemplary flow cytometric analysis of an ischemic hemisphere at 24 h after MCAO. **Vital brain cells can be clearly identified within the forward (FSC) / sideward (SSC) scatter plot. These cells can be further distinguished into a CD45-negative (i) and a CD45-positive fraction, the latter containing CD45low (ii) resting microglia and CD45high (iii) infiltrated leukocytes.

**Figure 5:**
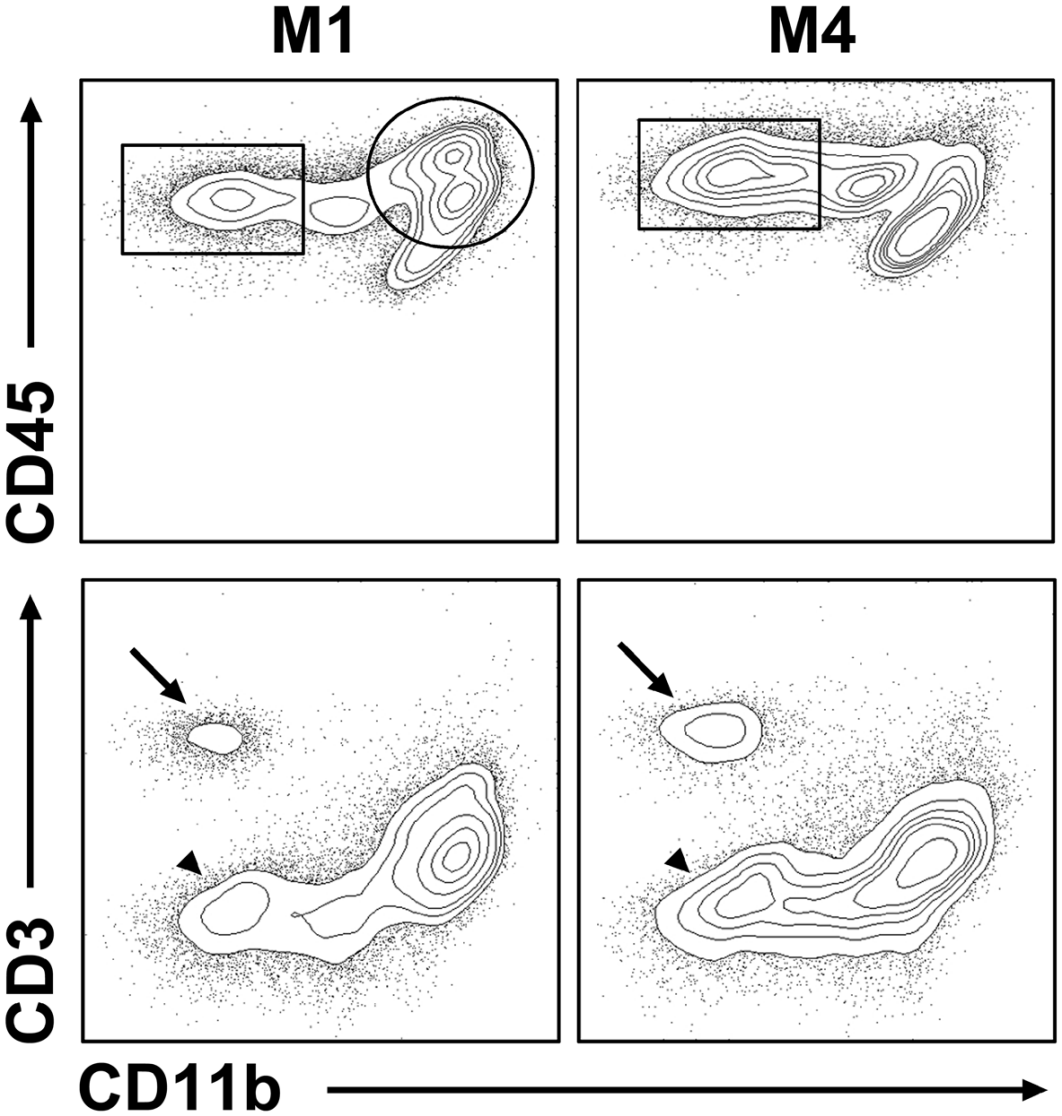
**Exemplary downstream analysis of immune cell subpopulations at day 1 (M1) and 4 (M4) after stroke.** At M1, isolated immune cell suspensions contain mainly CD11b-positive/CD45intermediate and CD45high myeloid cells (circle) whereas proportionally more CD11b-negative/CD45high non-myeloid cells infiltrate the ischemic brain at M4 (rectangular). These non-myeloid cells can be further differentiated into CD3-positive T-cells (arrow) and CD3-negative cells (B-cells and other lymphoid cells; arrowhead).

The methodology described above yields reliable and reproducible results for both the amount of viable cells per hemisphere and the proportion of CD45-positive cells within the lifegate. Comparable viable cell recoveries were measured in both healthy control animals and animals that were subjected to sham surgery (control: 1.7 ± 0.6 x 10E5 cells; sham at day 1: 2.0 ± 0.6 x 10E5 cells; sham at day 4: 1.8 ± 0.4 x 10E5 cells). In contrast, cell counts were almost twice as much in animals that underwent experimental stroke, irrespective of the time point investigated (stroke at day 1: 3.8 ± 1 x 10E5 cells; stroke at day 4: 4.2 ± 0.9 x 10E5 cells). The proportion of CD45-positive cells within the lifegate was consistent in all experimental groups (68 ± 10 %; Figure 3A) indicating the protocols reliability independent from the condition of the brain or the time point of investigation. The amount of CD45-positive cells hence increased significantly in consequence of brain ischemia (Figure 3B). CD45-positive cells could be further subdivided into CD45low cells that represent resting microglia and CD45high cells that contain activated microglia and peripheral leukocytes [16] (Figure 4). However, inflammatory cell infiltration after stroke is a highly dynamic process [15] and the composition of leukocyte subpopulations may significantly depend upon the time points of observation (Figure 5).

### Advantages

• Highly reproducible results as shown by low variability of viable cell yield and proportion of CD45+ cells within the lifegate

• Purified immune cell suspension with low contamination of debris and unwanted cells

• Cell yield per hemisphere is sufficient to either measure up to three different flow panels or subject cells to additional experimental procedures

### Disadvantages

• Time consuming protocol (4 to 5 hours until purified cell suspension is obtained)

• Multi-step protocol and technically complex procedures (especially trituration and setup of multilayer gradients), requires significant training to produce reliable results

## Conclusions

The assessment of post-ischemic inflammation in the brain by multicolour flow cytometry not only allows a deeper understanding of immunological processes that substantially influence the development and outcome, but may as well be a promising tool to detect new therapeutic approaches to selectively stimulate beneficial pathways in the subacute course of stroke pathology. Therefore our study provides the fundamental methodology to implement such analyses onto the laboratory rat, which offers good options to combine functional testing with neuroimmunological data.

## Competing interests

The authors declare that they have no competing interests.

## Authors’ contributions

KM, TS, JB and DCW designed the study; KM performed the experiments; TS and JB supervised the experiments; KM and DCW wrote the manuscript. All authors read and approved the final manuscript.
